# Cytomegalovirus Infection in Adult Patients with Inflammatory Bowel Disease: A Literature Review

**DOI:** 10.34172/aim.2024.40

**Published:** 2024-05-01

**Authors:** Zahra Momayaz Sanat, Zeinab Siami, Sudabeh Alatab, Homayoon Vahedi, Zeinab Fanni

**Affiliations:** ^1^Digestive Disease Research Center, Digestive Disease Research Institute, Tehran University of Medical Sciences, Tehran, Iran; ^2^Department of Infectious Disease, School of Medicine, Ziaeian hospital, Tehran University of Medical Sciences, Tehran, Iran; ^3^Ziaeian Hospital, Tehran university of Medical Sciences, Tehran, Iran

**Keywords:** CMV-associated colitis, Cytomegalovirus infection, Inflammation, Inflammatory bowel disease, Treatment, Ulcerative colitis

## Abstract

Human cytomegalovirus (HCMV) is classified within the *Herpesvirales* order and is prevalent in 50%‒80% of the general population. Most carriers experience this infection without noticeable clinical symptoms. HCMV causes a lifelong latent infection that can be reactivated due to immune disorders and inflammation. The reactivation of HCMV becomes particularly significant when it coincides with inflammatory bowel disease (IBD). While cytomegalovirus (CMV) colitis in IBD patients was identified years ago, the role of CMV in triggering flare-ups, acute severe colitis, treatment resistance, and other outcomes in IBD patients experiencing CMV reactivation remains a subject of ongoing debate. In this review, we aim to address an updated insight into aspects related to the CMV colitis in IBD patients including epidemiology, risk factors, clinical features, diagnostic tests, histology, place of immunosuppressants and indications for antiviral treatment. We suggest for personalized and thorough assessment based on the disease phase and colitis severity when prescribing drugs to these patients. Furthermore, we emphasize the importance of regular patient follow-up to monitor drug side effects, ensuring treatment success, and minimizing the risk of colectomy.

## Introduction

 Human cytomegalovirus (HCMV) belongs to the human *Herpesviridae* family. It is a common viral infection with a seroprevalence of between 40 and 100% in the general population.^1^ In healthy people, HCMV usually causes a mild and self-limited disease. However, HCMV has the ability to persistently integrate into the DNA of host cells following the initial infection and be reactivated in response to several stimuli.^2,3^

 HCMV infection is of particular interest in patients with inflammatory bowel diseases (IBD). IBD patients are predisposed to latent CMV reactivation, because of the presence of chronic inflammation in the colon, inadequate nutritional intake, and impairment of natural killer cells function as well as receiving long-term maintenance of immunosuppression therapy.^4^

 Unrecognized CMV infection in IBD patients may result in fulminant disease, requiring colectomy or even death.^1^ The debate is still unsettles on the significance of CMV colitis among IBD patients, especially in aspects such as the role of HCMV in induction of flare or worsening of ulcerative colitis (UC) severity, alteration of resistance to treatment, and its effects on disease outcomes.^5^ Our aim in this review is to present the available data on role of HCMV in IBD and also to review the risk factors, diagnosis and treatment of HCMV infection.

## Structure, Transmission and Infection

 HCMV is a member of the human *Herpesviridae* family, encompassing viruses such as Epstein-Barr virus (EBV), Herpes Simplex virus types 1 and 2 (HSV-1,2), Varicella-Zoster virus, and Human Herpes virus types 6 and 7 (HHV-6,7). Characterized by an icosahedral shape, the virus has a diameter ranging from 150 to 200 nm, comprising four essential structural components: an outer lipid envelope, tegument, a nucleocapsid, and an internal nucleoprotein core housing its genome.^6,7^

 Transmission can occur through close personal exposure to bodily fluids, via organ transplantation and from mother to fetus during pregnancy, causing non-genetic congenital sensorineural hearing loss and neurological damage.^8^

 Upon initial infection, the HCMV genome is expressed and replicated in three sequential steps of immediate-early, early and late.^9^ Following primary HCMV infection, the virus uses several mechanisms to avoid detection by the immune system and establishes a lifelong latent infection in host cells. During the latency phase, viral DNA persists as an episome in the nucleus without integration into the cellular genome.^10-12^

 Reactivation of the latent virus can occur in response to several stimuli, such as immunosuppressant therapy, infection, signiﬁcant stress or chronic inflammation.^13^ While both acute infection and subsequent reactivation of the virus are generally asymptomatic and self-limited in an immunocompetent individual, in immunocompromised patients, such as those with IBD, primary infection or reactivation is associated with significant complications and morbidities.^14^

 In this regard, it is essential to distinguish HCMV infection from the HCMV disease. CMV infection can be latent (presence of CMV viral DNA without detectable replication) or active (evidence of active viral replication or remarkable elevation in HCMV-speciﬁc antibodies without symptoms), whereas the CMV disease is the presence of clinical overt symptoms concomitant with CMV infection.^15^

## Epidemiology

 The reported prevalence of CMV colitis varies significantly across studies, primarily due to differences in the applied definitions, diagnostic criteria involving histological and/or serological markers, and the specific characteristics of the studied populations. Typically, the highest prevalence rates are observed in studies that define CMV infection based on a positive serum polymerase chain reaction (PCR). In instances where CMV intestinal disease is diagnosed, studies utilizing tissue PCR with a detection threshold exceeding 10 copies/mg tissue tend to report the highest prevalence of CMV infection.^16^

 The seroprevalence of CMV infection is similar between IBD and non-IBD subjects. A recent meta-analysis, including 1168 IBD patients from18 studies reported that latent CMV infection rates, assessed by HCMV IgG tests, was 69.6% among IBD patients compared to 51.8% in the control group.^17^ Prevalence studies showed that while CMV seropositivity in CD patients is similar to UC subjects, the frequency of both CMV infection and CMV intestinal disease reactivation is much lower in CD compared to UC patients, making the HCMV to be an unlikely etiology for clinical evolution.^18^ The probable reason might be attributed to predominant production of tumour necrosis factor alpha (TNF-α) in UC which is believed to promote activation of CMV. In contrast, CD is considered a Th1-type inflammatory process with high production of interferon-γ (IFNγ) from CD4^+^ T, a factor that is supposed to suppress CMV reactivation.^19^

 The prevalence of associated CMV colitis ranges from 10% to 17% in patients with severe colitis. Lopes and colleagues followed 95 endoscopically active IBD subjects and reported that 12.1% of them had positive tissue PCR–CMV.^19^ A cross-sectional study from Iran showed that 7% of UC patients were positive for tissue PCR–CMV.^20^ Roblin and colleagues conducted an assessment involving 60 adult IBD patients experiencing a moderate to severe flare. Their findings revealed that the prevalence of CMV infection at the tissue level was greater among patients with UC, with 38.1% (16 out of 42) affected, compared to those with CD, where the prevalence was 11.1% (2 out of 18).^21^ Studies show that the prevalence of CMV colitis is rising when patients present with acute sever colitis. In a prospective study from the USA, Kim and colleagues included 122 UC patients and reported the CMV positive rate (by immunohistochemistry staining for CMV antigen) at 21%–34% in patients with acute severe colitis, at 33–36% in corticosteroid-refractory cases, and at 10% in active UC.^22^ In a multicenter, prospective Korean study, CMV infections were identified in 43% of patients with moderate-to-severe active disease and increased to 67% in those who were corticosteroid-refractory.^23^

## Risk Factor

 In adult IBD patients, several risk factors are suggested to be associated with CMV colitis. Older age and older age of UC onset have been suggested by studies as risk factors. Gauss et al^24^ and McCurdy et al^25^ showed a higher risk of CMV colitis in IBD patients with age higher than 30 years. A recent meta-analysis proposed that UC patients with a later age of disease onset are more likely to have CMV reactivation.^26^

 Some studies have shown that CMV colitis is more frequent in patients with shorter IBD duration. Gauss et al indicated the association of occurrence of CMV infection with IBD duration less than 5 years^24^ however, this factor is still subject to debate as a systematic review including 2099 UC patients could not establish such a relation.^26^

 Disease severity and disease extension are among other risk factors of CMV colitis.^27^ In the setting of acute severe colitis, Lee and colleagues showed a 1.5-time higher risk of CMV infection for each point increase in Mayo score.^28^ This finding was supported by observations from a meta-analysis performed by Qin et al in which the risk of CMV reactivation in patients with severe UC was 1.5 times higher than that of patients with mild-to-moderate UC.^26^ Patients with extensive involvement of the colon (pancolitis) are also at increased risk of CMV infection (almost 2 times) compared to those with lesions limited to the left colon.^26,29^

 In addition to disease features, immunosuppression therapy has a significant role in the risk of CMV reactivation. A recent meta-analysis showed that glucocorticoid therapy in various forms escalates the risk of CMV reactivation by 4.17 times (95% CI: 3.07 to 5.66, P = 0.001).^26^ However, the debate still continues on the relation between glucocorticoid doses and CMV risk. Matsuoka et al declared the cumulative glucocorticoid usage of higher than 400 mg within one month as a risk factor^30^ while Lee and colleagues showed the higher risk of CMV with daily average glucocorticoid use of more than 40 mg for one month.^28^ Azathioprine, calcineurin inhibitors (such as cyclosporine A and tacrolimus), and simultaneous use of more than two lines of immunosuppressive drugs are identified as risk factors for CMV colitis.^25,28,29,31^

 Notably, the majority of the literature evidence demonstrated that TNF antagonists are not a risk factor for CMV infection, which might be pointing out the stimulatory effects of TNF-α on reactivation of CMV and therefore, the inhibitory effects of TNF-α antagonists on CMV reactivation.^32^

 Sandborn and colleagues pooled the available data on the safety of tofacitinib for treatment of moderate to severe UC and reported only one case of CMV colitis.^33^ These data suggest that tofacitinib and this treatment is safe regarding the risk of CMV infection.

 Some recent studies suggest that vedolizumab, a gut-selective integrin inhibitor that targets the homing of α4β7 lymphocytes, might increase the risk of developing the CMV disease,^34^ although further studies are needed to confirm these preliminary observations.

## Factors Contributing to Colonic Reactivation of CMV in IBD

###  Inflammation

 Typically following primary infection, CMV remains dormant until it becomes reactivated preferentially in the inﬂamed colonic mucosa of active IBD patients.^35^

 In IBD patients, there is a disturbance in both innate and adaptive immune responses, leading to the localized upregulation of various proinflammatory cytokines, including TNF-α, IFNγ, IL-6, and IL-23.^36,37^ In the setting of a proinflammatory environment and in response to inflammatory cytokines, particularly TNF-α, infected monocytes are attracted to inflamed areas, where they undergo transformation into actively replicating macrophages and subsequently generate viral infectious particles.^38-40^ The coupling of TNF-α to its receptor initiates a cascade with increased production of protein kinase C and activation of NF-κB pathway which in combination with relative T-cell dysfunction, stimulates the transcription of the CMV immediate early genes, loss of control of CMV latency and, thus, viral replication.^41,42^ Proinflammatory prostaglandins, stress catecholamines, epinephrine, and norepinephrine also activate the expression of immediate early genes.^42^ This process is further exacerbated by the interaction between activated monocytes, colonic endothelial cells and T cells with a final result of increased proinflammatory cytokines production, deterioration in the clinical condition and, in some cases, resistance to treatment.^43,44^

###  Immunosuppressive Drugs

 Systemic administration of certain immunosuppressive drugs in IBD patients could stimulate CMV reactivation. Several studies have indicated an increased risk of CMV colitis in IBD patients exposed to steroid therapy.^24,31,45^ *In-vitro* studies show that corticosteroids could induce CMV replication.^46^ Corticosteroids may play a role in triggering viral replication by suppressing the immune cell effector functions, including those of natural killer (NK) cells.^47,48^ Moreover the immunosuppressive drugs might impair T lymphocytes function, resulting in reactivation of the virus.^41^ Therefore, in the context of UC inflammation, administration of corticosteroids and immunosuppressive can potentially trigger the reactivation of the virus and enhance the migration of CMV-infected monocytes and macrophages into inflamed tissues. Once activated, CMV can infect various cell types, including epithelial, vascular endothelial, and interstitial cells, throughout the differentiation and stimulation phases. This leads to an uncontrolled inflammatory cycle characterized by increased production of proinflammatory cytokines, such as IL-6 and TNF-α, fostering more virus replication. Consequently, a detrimental cycle is established, exacerbating intestinal inflammation and contributing to a worsening clinical state.^35,43,44^

###  Clinical and Endoscopic Features

 Symptoms of CMV colitis are nonspecific and may mimic symptoms of IBD flares.^49^ Symptoms may include diarrhea, bloody stool, crampy stomach pain, rectal urgency, and tenesmus as well as systemic symptoms like fever, anorexia, malaise, nausea, vomiting, and weight loss.^50-52^ Hematochezia (bloody stools) and diarrhea are recognized as the two most common symptoms.^51^ The biochemical abnormality may include: elevated C-reactive protein (CRP) levels, low WBC counts, and low hemoglobin and albumin levels as well as thrombocytopenia.^50^

 Potentially fatal complication such as massive colonic bleeding, megacolon, fulminant colitis, and perforation (about 1% of cases) could occur in these patients.^53^ In a meta-analysis, positive CMV patients, had higher chance of severe colitis (RR, 1.32; 95% CI, 1.04‒1.67), pancolitis (RR, 1.31; 95% CI; 1.01‒1.72) and surgery (RR, 2.13; 95% CI, 1.03‒4.40).^54^ In cases where patients fail to show improvement with steroid therapy, suspicion of CMV colitis is warranted.^55^ Studies suggest that CMV infection may elevate the risk of steroid resistance in IBD patients nearly twofold, with a pooled RR of 2.12 (95% CI = 1.72–2.61) and a corresponding resistance rate of 70%.^56^

 Whether the CMV infection can affect the in-hospital mortality rate is controversial, with some studies showing a higher rate of mortality (up to 7 times higher),^57^ while others could not establish such a strong relation.^14^ Perhaps the tissue viral load is an important factor for determining the poorer outcome in these patients.

**Figure 1:**
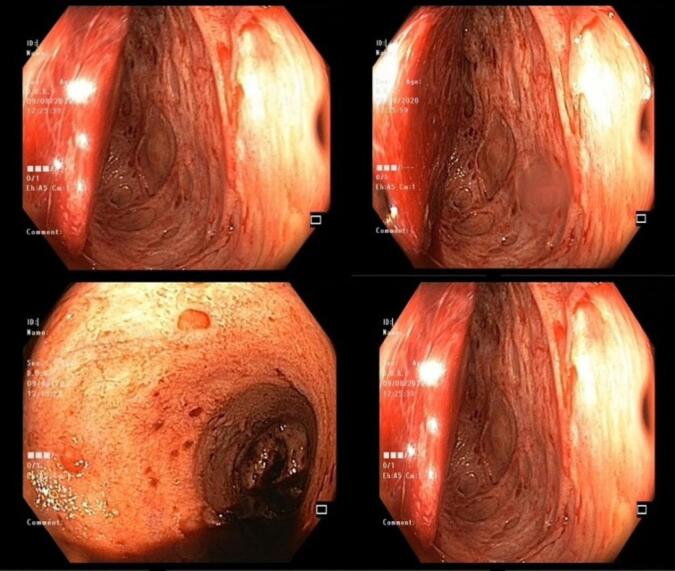
Endoscopy Findings in CMV Colitis

 Like CMV colitis symptoms, the endoscopic findings are nonspecific.^29^ Diffuse erythema, hemorrhagic patches, ischemia, superficial erosions, ulcers, strictures, polyploids, pseudomembranes, and pseudotumors are some of the nonspecific endoscopic findings, as are conspicuous, thick, pseudomembrane-like exudate coverings (Figure 1). Irregular ulceration, map-like appearance, ulcerations with a well-defined, punched-out appearance, and longitudinal ulceration are among some findings that have been reported to be associated with CMV colitis.^58-60^ Diagnosis of CMV colitis requires histological evaluation of biopsy tissue. Because CMV inclusion body (histological pathognomonic marker) is more prevalent at the base of ulcers, mucosal biopsies should be preferably taken from the base and edge of the ulcer.^61^ To maintain a high likelihood of identifying CMV in colonic tissue, complete colonoscopy with a minimum of 11 biopsies from the entire colon in UC and 16 biopsies in CD are required.^59,60^

## Diagnosis of CMV Infection

 Presently, various techniques are available for diagnosing intestinal CMV infection, including serology, viral culture, CMV antigen testing, histology, and CMV DNA testing in both blood and intestinal tissue (Table 1). Studies predominantly support the notion that detecting CMV in colonic tissue has more clinical relevance than in the blood. Consensus among numerous studies suggests that histological diagnosis should be regarded as the “gold standard” for identifying CMV disease. The challenge lies in distinguishing between an acute flare of UC and CMV colitis. Current guidelines from the European Crohn’s and Colitis Organization (ECCO) now recommend screening for CMV, particularly in cases of treatment-refractory or severe relapse.^62^

**Table 1:** Characteristics of Diagnostic Tests for CMV Colitis

**Diagnostic Test **	**Advantages**	**Disadvantages**
CMV IgG Class Antibodies	Confirms previous CMV exposure Helps identify risk for CMV colitis	Does not provide information about intestinal disease Does not reflect reactivation
CMV IgM class antibodies	- Confirms acute infection or reactivation	Does not provide information about intestinal disease
Antigen (pp65) detection assay	Short turnaround time (24 hours) High specificity for CMV colitis Helps predict clinical course of CMV colitis	Relatively low sensitivity for CMV colitis
CMV DNA (PCR in blood)	No endoscopy required	No established cutoff for the diagnosis of CMV colitis
CMV DNA (PCR in tissue)	Very high sensitivity for CMV in the colon	Low specificity, uncertain clinical significance No established cutoff
CMV DNA (PCR in stool)	No endoscopy required	Limited experience with the method
Viral culture	High sensitivity and specificity for CMV	Long time to obtain results (2–4 weeks)
Histological examination (H/E staining)	High specificity for CMV colitis	Invasive Time-consuming Low sensitivity for CMV colitis Requires many tissue samples Requires skilled pathologist
Histological examination (IHC staining)	More sensitive than H/E High specificity for CMV colitis	Invasive Time-consuming Requires many tissue samples Requires skilled pathologist

CMV, cytomegalovirus; IgG, immunoglobulin G; IgM, immunoglobulin M; PCR, polymerase chain reaction; HE, hematoxylin and eosin; IHC, immunohistochemistry.

## Serology Test

 Serology has limited value for CMV colitis, mostly because of the high seroprevalence of CMV in IBD patients. The serum levels of neither anti-CMV IgG Ab or IgM Ab have any clinical role in active CMV colitis and should not be measured unless viremia (uncommon in UC patients) is suspected.^18^ Sensitivity of IgM serology in detecting CMV disease is around 15%‒60%; however, if the assays detect CMV pp65 antigen in circulating leukocytes, higher sensitivities (60%–100%) and specificities (83%–100%) can be reached.^38^ Detecting IgG Ab against CMV can be useful for targeting patients at risk of reactivation. Some studies suggest that a significant ( > 4-fold) increase in anti-CMV IgG Ab could indicate infection,^9^ but it needs paired measurement of Ab in 2‒4-week intervals and most of the time there are no recent prior IgG levels available with which to make the comparison.^9,63^

## Culture

 CMV culture involves isolation of the virus from cultured cells and its confirmation by Immunofluorescence assays. CMV blood culture has relative low disease detection sensitivity (45‒78%), requires a long incubation period of 3 weeks, and has high false-negative results.^4^ However, a positive blood culture is very specific (89%‒100%) for and predictive of the CMV disease.^16^

## Antigen Test

 The CMV antigenemia assay stains the structural virus protein pp65 in the patient’s peripheral-blood mononuclear cells, via staining with immunoﬂuorescent pp65-speciﬁc monoclonal Ab,^9^ and then reports the number of positive cells per fixed number of leukocytes.^64^ The test has a sensitivity of 60% to 100% and a specificity of 83% to 100%.^35^ One limitation of the testing process is its labor-intensive nature, coupled with its dependency on the operator, and the requirement for sample processing within a narrow window of 6 to 8 hours. Additionally, a positive result lacks gut specificity and does not provide differentiation between latent infection and active disease.^35^

## Histology

 Histological diagnosis is currently the most reliable method for detecting clinically relevant CMV disease and therefore, early colonoscopy is considered necessary for diagnosis.^65^ Virus in the colonic tissue could be detected by the following techniques: hematoxylin and eosin (H&E) staining, IHC and quantitative polymerase chain reaction (qPCR, colonic CMV replication).^66^ International guidelines recommend ICHor tissue PCR as the accepted method of CMV detection in colonic tissue.^27^

###  Hematoxylin and eosin staining 

 The typical histological feature of CMV infection using conventional H&E staining is owl’s eye appearance.^58^ This histological appearance has high specificity for diagnosis of CMV colitis (92‒100%) and it is pathognomonic of tissue infection. However, its sensitivity is low around 10 to 87%,^35^ as owl’s eye appearance does not always exist or is detected by pathologists. Up to 37% of patients with CMV colitis fail to show any inclusion.^4^ Therefore, H&E histological staining is insufficient for diagnosis of CMV colitis because of the high rate of false negative biopsies.^4,58^

###  Immunohistochemistry

 CMV-specific IHC staining labels infected cells using monoclonal Abs directed against one of the CMV immediate early Ags.^4,67^ This technique allows a semi-quantification of CMV infection by reporting the number of positive-colored nuclei/field.^38^ IHC has a higher sensitivity (78-93%) and diagnostic specificity (92‒100%) compared to H&E staining for colonic CMV.^4,67^ Kredel et al showed that IHC has 67% sensitivity for diagnosis of CMV colitis compared to 17% by H&E staining.^68^

 IHC should be performed when CMV colitis is clinically suspected. IHC is also important in evaluation of IBD patients with severe disease before treatment modification.^69^ The inclusions in IHC are mainly seen within endothelial cells.^17^ ECCO guidelines 2017 recommend H&E staining for identification of CMV inclusions and preferably also IHC and/or quantitative tissue PCR, but stress that detecting several intra-nuclear inclusions, rather than occasional cells, are clinically significant.^69^ While previous studies reported no association between the density of positive cells in IHC with the number of viral load copies by PCR,^70^ Zidar and colleagues in a more recent study showed a correlation between IHC and CMV–PCR load.^71^

###  Polymerase Chain Reaction

 Technically, qualitative and quantitative PCR for CMV DNA can be performed on peripheral blood, colonic tissue, or stool,^35^ but it has low sensitivity (44%) and specificity (88%).^55^ The PCR for CMV detection on colonic tissue is more sensitive than IHC (92%–96%) and its result is independent of the observer.^27,35,67,72^ This test also has high specificity (40-100%); however, the tissue- PCR assay preferably should be done on fresh rather than formalin-fixed samples, as the fixation process reduces the nucleic acid integrity and sensitivity of PCR.^27^ Quantitative PCR, rather than qualitative PCR, may be more accurate, as the viral load and not only the presence of CMV-DNA, has been associated with CMV colitis and response to anti-viral therapy.^73^ In a study by Roblin and colleagues, the assessment of intestinal tissue CMV DNA load in 42 hospitalized UC patients experiencing acute flare-ups revealed that a CMV DNA load exceeding 250 copies/mg of tissue serves as a predictive indicator for resistance to immunosuppressive therapies such as steroids, infliximab, and cyclosporine.^21^ Another study conducted by Ciccocioppo et al, involving a cohort of 40 IBD patients, identified a significant association between a DNA peak value equal to or greater than 10^3^ copies/10^5^ cells and treatment refractoriness.^74^ Therefore, it is recommended that the use of PCR should be limited to those patients with negative IHC but strong clinical suspicion of CMV reactivation.^70^

## Anti-viral Treatment

 Currently, there is no consensus on the therapeutic approach to active CMV infection in IBD patients. Decision should be made based on a thorough and comprehensive individualized risk-benefit assessment.^35^ Antiviral therapy could be associated with considerable side effects and it may not be effective in some patients.^75^ The American College of Gastroenterology (ACG) and ECCO recommend antiviral therapy in moderate to severe colitis patients whose histological examination of mucosal tissue reveals high-grade CMV density, or those who have corticosteroid-refractory disease or are corticosteroid-dependent.^35^

 The drug of choice for CMV colitis in adults is intravenous ganciclovir administered at a dosage of 5 mg/kg twice daily (BID) for a duration of 2 to 3 weeks.^76^ Based on ECCO guidelines, in the presence of an early clinical response, typically observed after 3 to 5 days of treatment, a transition can be made to oral valganciclovir. The recommended dosage for oral valganciclovir is 900 mg administered twice daily, and this oral therapy is continued for the remaining duration of the 2 to 3-week treatment course for CMV colitis in adults, although as inflammation of the gut may compromise drug absorption, risk of CMV reactivation should be considered.^69^ Valganciclovir is the pro-drug of ganciclovir and has superior oral absorption. The remission rates after anti-viral treatment in IBD–CMV infected patients is high, ranging from 67% to 100%.^67,77^ Factors such as baseline CMV DNA loads, kinetics of replication, and rate of viral decay may be involved in the rate of treatment response.^78^

 Myelotoxicity with features of bone marrow suppression (neutropenia, and thrombocytopenia) is a serious side effect of ganciclovir.^35^ Patients need regular monitoring of their blood cell counts throughout the treatment period. Additionally, common side effects include skin rash, low blood pressure, nausea, vomiting, and headaches.^55^

 Ganciclovir resistance should be considered if patients fail to respond to treatment. Mutations in the UL97 and UL54 genes are two underlying mechanisms for development of ganciclovir resistance. In case of resistance or intolerance, Foscarnet, or cidofovir may be used although they have a high risk of nephrotoxicity.^76^

## Place of Anti-viral Treatment

 The available data regarding the effect of antiviral therapy on achieving clinical improvement, mortality, colectomy rate and overall prognosis is inconclusive. Al-Zafiri and colleagues reviewed charts of emergency admitted patients with diagnoses of IBD flare and CMV over a 10-year period and found no significant difference between patients who received antiviral therapy compared to those who did not in terms of achieving clinical improvement, avoidance of colectomy, and death (64% versus 70%).^79^ Accordingly, in a systematic review, analysis of long-term colectomy rates in 110 patient from 6 studies showed no statistically significant difference between CMV-positive UC patient who received antiviral therapy compared to untreated groups (OR = 1.71; 95%CI: 0.71‒4.13).^80^ However, Shukla et al showed the significant beneficial effect of antiviral therapy on colectomy risk when introduced in steroid refractory UC patients presenting with flare-ups (OR 0.20; 95% CI 0.08‒0.49).^81^ Some studies propose that response rates and outcomes of patients might depend on the CMV viral concentration in the colonic tissue.^38^ In the investigation conducted by Jones and collaborators, individuals with high-grade CMV density ( ≥ 5 inclusions in any single fragment on IHC) and those with low-grade CMV density ( < 5 inclusions in any single fragment on IHC) both experienced advantages in terms of surgery-free survival outcome with antiviral treatment. However, the enhancement and postponement of surgery were more notable among patients with high-grade CMV density.^76^ It is noteworthy that the cut-off point to start antiviral treatment is an issue of debate and has not been standardized yet.

## Prognosis and outcome of CMV colitis on UC flares

 It is generally accepted that reactivation of CMV can trigger flare-ups, worsen mucosal damage, and reduce the duration of remission. Patients with UC who have CMV infection, particularly those compromised by corticosteroid therapy, tend to experience severe symptoms.^27^ Research has observed more adverse outcomes in IBD patients with positive CMV testing. These outcomes include higher rates of resistance to IBD therapy, increased complications leading to a higher colectomy rate, more urgent colectomies with extended postoperative hospitalization, more frequent postoperative complications, and even more deaths.^16,45,67^ Several studies support the idea that the presence of CMV in acute severe colitis serves as a negative prognostic marker, suggesting a more severe or refractory phenotype. These studies indicate that colectomy rates are higher in patients with CMV colitis compared to those without concurrent CMV.^35^ Older age, male gender, comorbidities, low albumin level, and tissue-CMV were associated with increased risk of colectomy.^79^ However, there is another emerging concept in IBD colitis named “innocent bystander” in which the CMV infection itself produces no significant detrimental effect on the course of IBD.^82^ Some studies have shown examples of active CMV infection in colitis patients who respond to steroid therapy without needing antivirals.^83^

 Individuals with CMV infection and acute severe colitis exhibit greater resistance to corticosteroid treatment compared to non-infected patients. Some studies report that between 25%‒81% of steroid-refractory UC patients have CMV.^77^ A systematic review demonstrated that the majority of patients with CMV infection and intestinal disease were refractory to steroid therapy.^16^ Another recent meta-analysis including 1306 patients, evaluated the rate of steroid resistance in IBD patients when CMV was detected by any method in two groups of CMV-positive and CMV-negative subjects. Their analysis showed a higher rate of steroid resistance in the positive group (52.9 vs 30.2%).^17^ However more research is needed to confirm whether CMV infection itself is related to steroid resistance or the presence of CMV infection in acute severe colitis is a poor prognostic marker indicative of a more severe or refractory phenotype.

 While HCMV infection can complicate both UC and CD, studies show a less important role for CMV infection in the clinical course of CD patients.^4,5^ Al-Zafiri and colleagues evaluated the impact of CMV disease on both CD and UC patients during a 10-year period and found that the rate of CMV in CD was significantly lower than UC patients (3.5% in CD vs 8.5% in UC, *P* = 0.012).^79^ However, similar to UC patients, older age and comorbidities contributed to the risk of CMV in CD patients.^79^

## How to Manage CMV Colitis with Immunosuppressive

 In general, it is not recommended to discontinue immunosuppressive therapy in IBD patients with CMV reactivation. According to ECCO guidelines, it is advised to continue immunosuppressive therapy alongside antiviral treatment in cases of subclinical or mildly symptomatic IBD. However, for patients with severe steroid-resistant CMV colitis, it is recommended to consider discontinuing or reducing immunomodulators until the symptoms of CMV colitis are under control, in addition to initiating antiviral therapy. In instances of systemic CMV reactivation (meningoencephalitis, pneumonitis, esophagitis, or hepatitis), immediate antiviral therapy is necessary, and all immunosuppressive therapies should be temporarily halted. The British Society of Gastroenterology has recently suggested that CMV colitis in hospitalized UC patients can be treated with intravenous ganciclovir while continuing conventional therapy with corticosteroids or using rescue medications such as infliximab or cyclosporine.^84^

 Cyclosporine use may increase the risk of CMV reactivation as cyclosporine induces its immunosuppressive effects through inhibition of T cell proliferation.^38^ The recommendation is against initiating cyclosporine in patients with severe colitis and concurrent CMV infection. However, recent findings from a study involving 119 patients with severe UC and CMV infection suggest that cyclosporine can be administered in combination with antiviral therapy. The study indicated no significant difference in short-term and long-term colectomy rates between patients who received ganciclovir alone and those who received a combination of ganciclovir and cyclosporine.^85^

 The main controversial medications are the corticosteroids. According to the experiment conducted by Ciccocioppo, steroids should be promptly tapered and discontinued. However, for patients with viral colitis (mucosal viral load ≥ 10^3^ /10^5^ cells) and those displaying reactivation of latent infection (viral load 10^2^ to 10^3^ copies/10^5^ cells), immunosuppressants and long-lasting biological agents are recommended to be maintained.^86^ On the other hand, Sager et al suggested continuing conventional corticosteroid therapy in conjunction with antiviral therapy.^67^

 Anti-TNF-α drugs, especially infliximab, are considered to have a lower risk of CMV reactivation than other immunosuppressants. Continuation/initiation of anti-TNF therapy is safe and acceptable, as it does not exacerbate the disease.^1^ Accordingly, Pillet et al showed that CMV DNA load did not become elevated during anti-TNF therapy.^87^ Murad et al suggested induction of remission with anti-TNF therapy in CMV colitis patients and then quick steroid tapering while continuing intravenous ganciclovir.^38^

 Even though its effectiveness has not been established in extensive patient cohorts, recent case studies have supported the use of vedolizumab in the treatment of steroid-resistant colitis with CMV reactivation.^88^

## Conclusion

 In IBD patients who present with worsening of their clinical pictures, CMV infection/reactivation should be suspected as delay in the diagnosis and appropriate management is associated with poor outcomes. The age of IBD onset, IBD duration, immunosuppressive drugs, and inflammation severity are the risk factors of CMV. There is an elaborate interplay between steroid resistance in IBD patients and CMV colitis which requires further studies. Among several ways for CMV colitis diagnosis, histological H&E and IHC stains in addition to tissue PCR seem to be the gold standards. Concerning the approach to CMV colitis, most studies propose that decision for antiviral therapy should be based on individualized assessment of the patient, severity of CMV colitis and steroid resistance. The decision should be made for the following items: start of antiviral therapy, adding anti-TNF agents as a step-up therapy, increasing immunosuppression, and/or stopping the corticosteroids.
